# Supplementary material for: Spatial coupling between DNA replication and mismatch repair in Caulobacter crescentus
Source: Nucleic Acids Res. 2021 Mar 2;49(6):3308–21. doi: 10.1093/nar/gkab112 (PMC8034640; doi:10.1093/nar/gkab112)
Supplement: gkab112_Supplemental_File [file gkab112_supplemental_file.pdf]

<u>Supplementary Information</u>

Spatial coupling between DNA replication and mismatch repair in *Caulobacter crescentus*

Tiancong Chai, Céline Terrettaz and Justine Collier

<u>Content:</u>

Supplementary Tables (page 2)

Table S1 (page 2)

Table S2 (page 3)

Table S3 (page 4)

Table S4 (page 5)

Table S5 (pages 6-7)

Supplementary Figures with legends (page 8)

Fig.S1 (page 8)

Fig.S2 (page 10)

Fig.S3 (page 11)

Fig.S4 (page 12)

Fig.S5 (page 13)

Fig.S6 (page 14)

Fig.S7 (page 15)

Fig.S8 (page 16)

Fig.S9 (page 17)

Supplementary Materials and Methods (page 18)

Supplementary References (page 20)

Supplementary Tables

Table S1: Oligonucleotides used in this study. Restriction enzyme recognition sites used for cloning purposes are underlined.

Name	Sequence (5'→3')	Use
**CT1**	GGTAAGCTTGGTCCGCGTGGGGATCTCTAC (HindIII)	Construction of pNPTS138-ΔmutS
**CT2**	CCCGGATCCGGGCGGCGTTAAGGAATTACAC (BamHI)	
**CT3**	CCCGGATCCTAGCGGGTACCCATATAGA (BamHI)	
**CT4**	CGGGCTAGCAACCCAGATCCCACAACGCATAC (NheI)	
**CT8**	CGGGCTAGCCGAGACGATGGATGACAAACACC (NheI)	Construction of pNPTS138-ΔmutL
**CT10**	GGTAAGCTTATGCCCATCCGCCGCCTGCCG (HindIII)	
**CT11**	CCCGGATCCCATGGCCTGGCGCTTGATCTCC (BamHI)	
**CT12**	CCCGGATCCTGAGGCCAGAGCGCCCCCTAC (BamHI)	
**TC46**	GTCGGATCCGCCTGATCGACAGGGCGAACCA (BamHI)	Construction of pNPTS138-ΔuvrD
**TC47**	CGGGAATTCCGCCGAAAAGCGAGCGTTCAGC (EcoRI)	
**TC63**	ATAAAGCTTATGTTTCCCGACACCGACTCGC (HindIII)	
**TC64**	CGTGGATCCGCGGTTCTTCCAGCCGTCGATG (BamHI)	
**TC18**	GCGAAGCTTGGTCCGCGTGGGGATCTCTA (HindIII)	Construction of pNPTS138-PmutS-YFP-MutS
**TC19**	GTGGGATCCCATGACGCCCGGAAACTACAG (BamHI)	
**TC20**	GAAGGATCCATGGTGAGCAAGGGCGAGGAG (BamHI)	
**TC21**	CTCGAATTCGGACAGCTCGACCGACGCCA (EcoRI)	
**TC42**	TGACTGACCGCGGCGGCGGCGGCGGCGTCCAGCTTGG	Construction of pNPTS138- MutS($_{849}$AAAAA$_{853}$) and pXYFPN4- MutS($_{849}$AAAAA$_{853}$)
**TC43**	CCAAGCTGGACGCCGCCGCCGCCGCCGCGGTCAGTCA	
**TC52**	AACAAGCTTGGTAAGTCGACCTTCCTGCGCC, HindIII	Construction of pNPTS138- MutS($_{849}$AAAAA$_{853}$)
**TC53**	GCGGAATTCGCTTGTAGGGCCGCAGGAACAG, EcoRI	
**TC34**	CGAAAGCTTCTTTCCCGGCGTCGTTGTGGA (HindIII)	Construction of pNPTS138-DnaQ(G13E)
**TC35**	TTTGGGTCAAACTCGGTGGTTTCG	
**TC36**	CGAAACCACCGAGTTTGACCCAAA	
**TC37**	GTGGGATCCGCCATGGCCCCTGTCGAATGG (BamHI)	
**TC14**	GGAAAGCTTCCAGCAGGCTCAGAATCCGAA (HindIII)	Construction of pNPTS138-PmutL-YFP-MutL
**TC15**	GCGGGATCCCATCAAGCGGACTTTCACGGG (BamHI)	
**TC16**	GACGGATCCATGGTGAGCAAGGGCGAGGA (BamHI)	
**TC17**	GGCGAATTCGGACTTCATGAATTTCAGGCG (EcoRI)	
**JC155**	CGGGTACCATGAACGCCCACGCCACGCCGACC (KpnI)	Construction of pXYFPN4-MutS, pXYFPN4-MutS($_{849}$AAAAA$_{853}$), pXYFPN4-MutS(F44A), pXYFPN4-MutS(K661M), pXYFPN4-MutS(E735A)
**JC156**	GGCTAGCCTAGGCCGTGAGCAGACCCTTAAGG (NheI)	
**TC27**	CAGCTCGTAGGCATCGCCCATGC	Construction of pXYFPN4-MutS(F44A)
**TC28**	GCATGGGCGATGCCTACGAGCTG	
**TC23**	GAAGGTCGACATACCGGCCATG	Construction of pXYFPN4-MutS(K661M)
**TC24**	CATGGCCGGTATGTCGACCTTC	
**TC38**	ATCCTGGACGCCATCGGCCGGG	Construction of pXYFPN4-MutS(E735A)
**TC39**	CCCGGCCGATGGCGTCCAGGAT	
**CoC-7_1**	TCGGTACCATGCCCATCCGCCGCCTGCCG (KpnI)	Construction of pXYFPN4-MutL, pXYFPN4-MutL($_{497}$ATLAAP$_{502}$), pXYFPN4-MutL(D472N) and pXYFPN4-MutL(D472N, $_{497}$ATLAAP$_{502}$)
**CoC-8_4**	GGAGCTCTGGCCTCACCGCCGCCCGAACAGC (SacI)	
**TC40**	ACCTCGGGGGCGGCCAGGGTGGCGCGGGTC	Construction of pXYFPN4- MutL($_{497}$ATLAAP$_{502}$) and pXYFPN4-MutL(D472N, $_{497}$ATLAAP$_{502}$)
**TC41**	GACCCGCGCCACCCTGGCCGCCCCCGAGGT	
**TC31**	CGCGTGCTGGTTGACAATGAC	Construction of pXYFPN4-MutL(D472N) and pXYFPN4-MutL(D472N, $_{497}$ATLAAP$_{502}$)
**TC32**	GGTCATTGTCAACCAGCACGCG	
**TC54**	TAGGTACCATGTTTCCCGACACCGACTCGC (KpnI)	Construction of pXYFPN4-UvrD
**TC55**	CCCGCTAGCTCAGGCCTTCTCGACGAAGCTG (NheI)	

Table S2: Plasmids used in this study.

Plasmid name	Description	Source
pNPTS138	Suicide vector carrying the *sacB* gene	D. Alley, unpublished
pNPTS138-ΔmutS	pNPTS138 derivative used to create the *ΔmutS* deletion strain	This study
pNPTS138-ΔmutL	pNPTS138 derivative used to create the *ΔmutL* deletion strain	This study
pNPTS138-ΔuvrD	pNPTS138 derivative used to create the *ΔuvrD* deletion strain	This study
pNPTS138-PmutS-YFP-MutS	pNPTS138 derivative used to replace *mutS* by *yfp-mutS* (under the control of native *mutS* promoter)	This study
pNPTS138-DnaQ(G13E)	To replace native *dnaQ (CCNA_00005)* with *dnaQ(G13E)* allele	This study
pNPTS138-MutS($_{849}$AAAAA$_{853}$)	To replace native *mutS* with the *mutS*($_{849}$AAAAA$_{853}$) allele	This study
pNPTS138-PmutL-YFP-MutL	pNPTS138 derivative used to replace *mutL* by *yfp-mutL* (under the control of native *mutL* promoter)	This study
pXYFPN4	For integrating constructs encoding N-terminal YFP fusions under the control of the native *xylX* promoter	(1)
pXYFPN4-MutS	*mutS* cloned into pXYFPN4	This study
pXYFPN4-MutS($_{849}$AAAAA$_{853}$)	*mutS*($_{849}$AAAAA$_{853}$) cloned into pXYFPN4	This study
pXYFPN4-MutS(F44A)	*mutS(F44A)* cloned into pXYFPN4	This study
pXYFPN4-MutS(K661M)	*mutS(K661M)* cloned into pXYFPN4	This study
pXYFPN4-MutS(E735A)	*mutS(E735A)* cloned into pXYFPN4	This study
pXYFPN4-MutL	*mutL* cloned into pXYFPN4	This study
pXYFPN4-MutL($_{497}$ATLAAP$_{502}$)	*mutL*($_{497}$ATLAAP$_{502}$) cloned into pXYFPN4	This study
pXYFPN4-MutL(D472N)	*mutL(D472N)* cloned into pXYFPN4	This study
pXYFPN4-MutL(D472N, $_{497}$ATLAAP$_{502}$)	*mutL*(D472N, $_{497}$ATLAAP$_{502}$) cloned into pXYFPN4	This study
pXYFPN4-UvrD	*uvrD* cloned into pXYFPN4	This study

Table S3: Strains used in this study.

Strain name	Genotype	Source
Escherichia coli		
TOP10	*F- mcrA Δ(mrr-hsdRMS-mcrBC) φ80lacZΔM15 ΔlacX74 nupG recA1 araD139 Δ(ara-leu)7697 galE15 galK16 rpsL(Str^R) endA1 λ$^-$*	Invitrogen (USA)
Caulobacter crescentus		
NA1000	Synchronizable derivative of wild-type strain CB15 (CB15N)	(2)
JC1427	NA1000 *ΔmutS*	This study
JC1426	NA1000 *ΔmutL*	This study
JC1575	NA1000 *ΔmutS ΔmutL*	This study
JC1847	NA1000 *ΔuvrD*	This study
JC1986	NA1000 *ΔuvrD ΔmutL*	This study
JC1784	NA1000 *YFP-mutS*	This study
JC1433	NA1000 Δ*mutS Pxyl::YFP-mutS*	This study
JC577	NA1000 *dnaN-CFP*	(3)
JC1625	NA1000 *dnaN-CFP* Δ*mutS Pxyl::YFP-mutS*	This study
JC1770	NA1000 Δ*mutS Pxyl::YFP-mutS*($_{849}$*AAAAA*$_{503}$)	This study
JC1799	NA1000 *mutS*($_{849}$*AAAAA*$_{503}$)	This study
JC1678	NA1000 *dnaQ(G13E)*	This study
JC1724	NA1000 Δ*mutS Pxyl::YFP-mutS* pNPTS138-DnaQ(G13E) integrated at *dnaQ* locus	This study
JC1666	NA1000 Δ*mutS Pxyl::YFP-mutS(F44A)*	This study
JC1669	NA1000 *dnaN-CFP* Δ*mutS Pxyl::YFP-mutS(F44A)*	This study
JC1665	NA1000 Δ*mutS Pxyl::YFP-mutS(K661M)*	This study
JC1739	NA1000 Δ*mutS Pxyl::YFP-mutS(E735A)*	This study
JC1668	NA1000 *dnaN-CFP* Δ*mutS Pxyl::YFP-mutS(K661M)*	This study
JC1740	NA1000 *dnaN-CFP* Δ*mutS Pxyl::YFP-mutS(E735A)*	This study
JC1769	NA1000 *YFP-mutL*	This study
JC1825	NA1000 Δ*mutL Pxyl::YFP-mutL*	This study
JC1812	NA1000 *dnaN-CFP* Δ*mutL Pxyl::YFP-mutL*	This study
JC1805	NA1000 *dnaN-CFP* Δ*mutS* Δ*mutL Pxyl::YFP-mutL*	This study
JC1749	NA1000 Δ*mutL Pxyl::YFP-mutL*($_{497}$*ATLAAP*$_{502}$)	This study
JC1750	NA1000 *dnaN-CFP* Δ*mutL Pxyl::YFP-mutL*($_{497}$*ATLAAP*$_{502}$)	This study
JC1667/JC1806	NA1000 Δ*mutL Pxyl::YFP-mutL(D472N)*	This study
JC1670	NA1000 *dnaN-CFP* Δ*mutL Pxy::YFP-mutL(D472N)*	This study
JC1894	NA1000 *dnaN-CFP* Δ*mutS* Δ*mutL Pxy::YFP-mutL(D472N)*	This study
JC1753	NA1000 *dnaN-CFP* Δ*mutL Pxyl::YFP-mutL(D472N,* $_{497}$*ATLAAP*$_{502}$)	This study
JC1870	NA1000 Δ*uvrD Pxyl::YFP-uvrD*	This study
JC1846	NA1000 *Pxyl::YFP-uvrD*	This study
JC2211	NA1000 *Pxyl::YFP-uvrD* pNPTS138-DnaQ(G13E) integrated at *dnaQ* locus	This study
JC1977	NA1000 Δ*mutS Pxyl::YFP-uvrD*	This study
JC1760	NA1000 *dnaN-CFP* Δ*mutS Pxyl::YFP-mutS ΔmutL*	This study

Table S4: Comparison of spontaneous mutation rates of *C. crescentus* strains used in this study. The symbols * and # indicate that the mutation rate is statistically significantly different or equivalent, respectively, to that of the NA1000 (WT) reference strain (for Zones A and D), of the JC1433 (*ΔmutS Pxyl::YFP-mutS)* strain (Zone B) or of the JC1825 *(ΔmutL Pxyl::YFP-mutL)* strain (Zone C). Student's *t*-tests were used to estimate statistical significance: differences were considered as significant (*) if P<0.05. The "relative mutation rate" value, facilitating comparisons, was calculated setting the spontaneous mutation rate of the NA1000 (WT) strain to a value of 1 (arbitrary units).

Zone	Strain number	Genotype (disturbed activity)	Number of Rif^R colonies * 10^{-9} total colonies [±standard deviation]	Relative mutation rate [% of DNA repair activity]
A	NA1000	WT	8.3 [±2.8]	1 [=100%]
	JC1427	*ΔmutS*	998.3 [±212.9]*	120.3 [=0%]
	JC1426	*ΔmutL*	916.7 [±312.4]*	110.4 [=0%]
	JC1575	*ΔmutS ΔmutL*	1152.7 [±423.1]*	138.9
	JC1847	*ΔuvrD*	540.1 [±95.0]*	65.1 [=0%]
	JC1986	*ΔuvrD ΔmutL*	1979.3 [±460.0]*	238.5
	JC577	*dnaN-CFP*	55.8 [±33.6]#	6.7
	JC1784	*YFP-mutS*	52.8 [±21.4]#	6.4 [95.5%]
	JC1799	*mutS*($_{849}$*AAAAA*$_{853}$) (β-clamp binding)	56.8 [±24.3]*	6.8 [95.1%]
	JC1678	*dnaQ(G13E)*	3973.3 [±1466.0]*	478.7
	JC1769	*YFP-mutL*	148.6 [±78.1]#	17.9 [84.6%]
	JC2212	*mutL*($_{497}$*ATLAAP*$_{502}$) (β-clamp binding)	932.7 [±315.6]*	112.4 [~0%]
B	JC1433	*ΔmutS Pxyl::YFP-mutS*	24.2 [±9.9]	2.9 [98.4%]
	JC1770	*ΔmutS Pxyl::YFP-mutS*($_{849}$*AAAAA*$_{853}$) (β-clamp binding)	45.0 [±21.2]#	5.4 [96.3%]
	JC1666	*ΔmutS Pxyl::YFP-mutS(F44A)* (mismatch recognition)	843.0 [±313.2]*	101.6 [15.7%]
	JC1665	*ΔmutS Pxyl::YFP-mutS(K661M)* (nucleotide binding and ATP hydrolysis)	792.3 [±75.0]*	95.5 [20.8%]
	JC1739	*ΔmutS Pxyl::YFP-mutS(E735A)* (ATP hydrolysis)	917.0 [±340.0]*	110.5 [8.2%]
C	JC1825	*ΔmutL Pxyl::YFP-mutL*	56.3 [±28.5]	6.8 [94.7%]
	JC1749	*ΔmutL Pxyl::YFP-mutL*($_{497}$*ATLAAP*$_{502}$) (β-clamp binding)	751.8 [±71.4]*	90.6 [17.2%]
	JC1667	*ΔmutL Pxyl::YFP-mutL(D472N)* (endonuclease)	904.3 [±204.2]*	109.0 [0.4%]
D	JC1870	*ΔuvrD Pxyl::YFP-uvrD*	102.5 [±39.5]#	12.3 [82.3%]

Table S5: Detection of distinct fluorescent foci (> 2-fold above average fluorescence of the cytoplasm) in (>1000) cells from strains used in this study and co-localization analyses.

	Strain name	Quantification	Average %	Standard deviations	Number of independent experiments
**Figure 2**	JC1433	Proportion of cells displaying a distinct focus (>2-fold above background)	69.6%	3.3%	3
	JC1770		0.1%	0.1%	
	JC1666		56%	2.8%	
	JC1665		47.8%	6.6%	
	JC1739		7%	3.0%	
**Figure 4**	JC1433	Proportion of cells displaying a distinct focus (>2-fold above background)	69.6%	3.3%	3
	JC1724		69.1%	5.6%	
	JC1825		32.4%	1.6%	
	JC1845		34.1%	3.5%	
**Figure 5**	JC1825	Proportion of cells displaying a distinct focus (>2-fold above background)	32.4%	1.6%	3
	JC1749		0.1%	0.2%	
	JC1667		49.8%	4.2%	
**Figure 6**	JC1812	Proportion of cells displaying a distinct DnaN-CFP focus (>2-fold above background)	75.1%	3.4%	3
	JC1750		59.4%	3.8%	
	JC1670		74.5%	2.4%	
	JC1753		58.3%	4.4%	
	JC1812	Proportion of cells displaying a distinct YFP-MutL focus (>2-fold above background)	45.1%	7.9%	
	JC1750		0.2%	0.3%	
	JC1670		63.8%	4.9%	
	JC1753		5.0%	1.1%	
	JC1812	Proportion of distinct YFP-MutL foci (>2-fold above background) that are co-localized with DnaN-CFP foci	97%	0.5%	
	JC1750		<0.1%	<0.1%	
	JC1670		97%	1.1%	
	JC1753		16%	5.4%	
**Figure 7**	JC1946	Proportion of cells displaying a distinct focus (>2-fold above background)	3.3%	1.8%	3
	JC2211		3.0%	1.2%	
	JC1977		1.7%	0.8%	
**Figure S4**	JC1625	Proportion of cells displaying a distinct DnaN-CFP focus (>2-fold above background)	66.1%	6.2%	3
	JC1771		54.2%	4.2%	
	JC1669		68.4%	3.1%	
	JC1668		67.4%	3.6%	
	JC1740		55.3%	4.6%	
	JC1625	Proportion of cells displaying a distinct YFP-MutS focus (>2-fold above background)	22.4%	3.6%	
	JC1771		0.8%	0.5%	
	JC1669		20.6%	2.0%	
	JC1668		2.6%	1.7%	
	JC1740		5.4%	2.0%	
	JC1625	Proportion of distinct YFP-MutS foci (>2-fold above background) that are co-localized with DnaN-CFP foci	96%	3%	
	JC1771		21%	18%*	
	JC1669		94%	3%	
	JC1668		79%	7%**	
	JC1740		83%	13%**	
**Figure S5**	JC577	Proportion of cells displaying a distinct DnaN-CFP focus (>2-fold above background)	57% without novobiocin	2.5%	3
			14% with novobiocin	1.7%	

	JC1433	Proportion of cells displaying a distinct YFP-MutS focus (>2-fold above background)	55% without novobiocin	7.4%	
			1% with novobiocin	0.4%	
	JC1825	Proportion of cells displaying a distinct YFP-MutL focus (>2-fold above background)	16% without novobiocin	1.8%	
			0.1% with novobiocin	0%	
**Figure S7**	JC1812	Proportion of cells displaying a distinct YFP-MutL focus (>2-fold above background)	45.1%	7.9%	3
	JC1805		32.2%	4.3%	
**Figure S8**	JC1670	Proportion of cells displaying a distinct YFP-MutL focus (>2-fold above background)	39.0%	10.9%	3
	JC1894		31.5%	4.0%	
**Figure S9**	JC1625	Proportion of cells displaying a distinct DnaN-CFP focus (>2-fold above background)	66.1%	6.2%	3
	JC1760		55.8%	2.7%	
	JC1625	Proportion of cells displaying a distinct YFP-MutS focus (>2-fold above background)	22.4%	3.6%	
	JC1760		15.0%	4.1%	
	JC1625	Proportion of distinct YFP-MutS foci (>2-fold above background) that are co-localized with DnaN-CFP foci	96%	3.4%	
	JC1760		92%	1.3%	

*Note that this standard deviation is unusually high, due to the fact that only very few cells (0.1%, as shown in Fig.2B) displayed distinct YFP-MutS($_{849}$AAAAA$_{853}$) foci; then, colocalization analyses were done on an only limited number of cells.

**Note that this standard deviation is higher than usually expected, due to the fact that low proportions of cells (2.6%/5.4%) displayed distinct YFP-MutS foci; then, colocalization analyses were done on a limited number of cells.

Supplementary Figures:

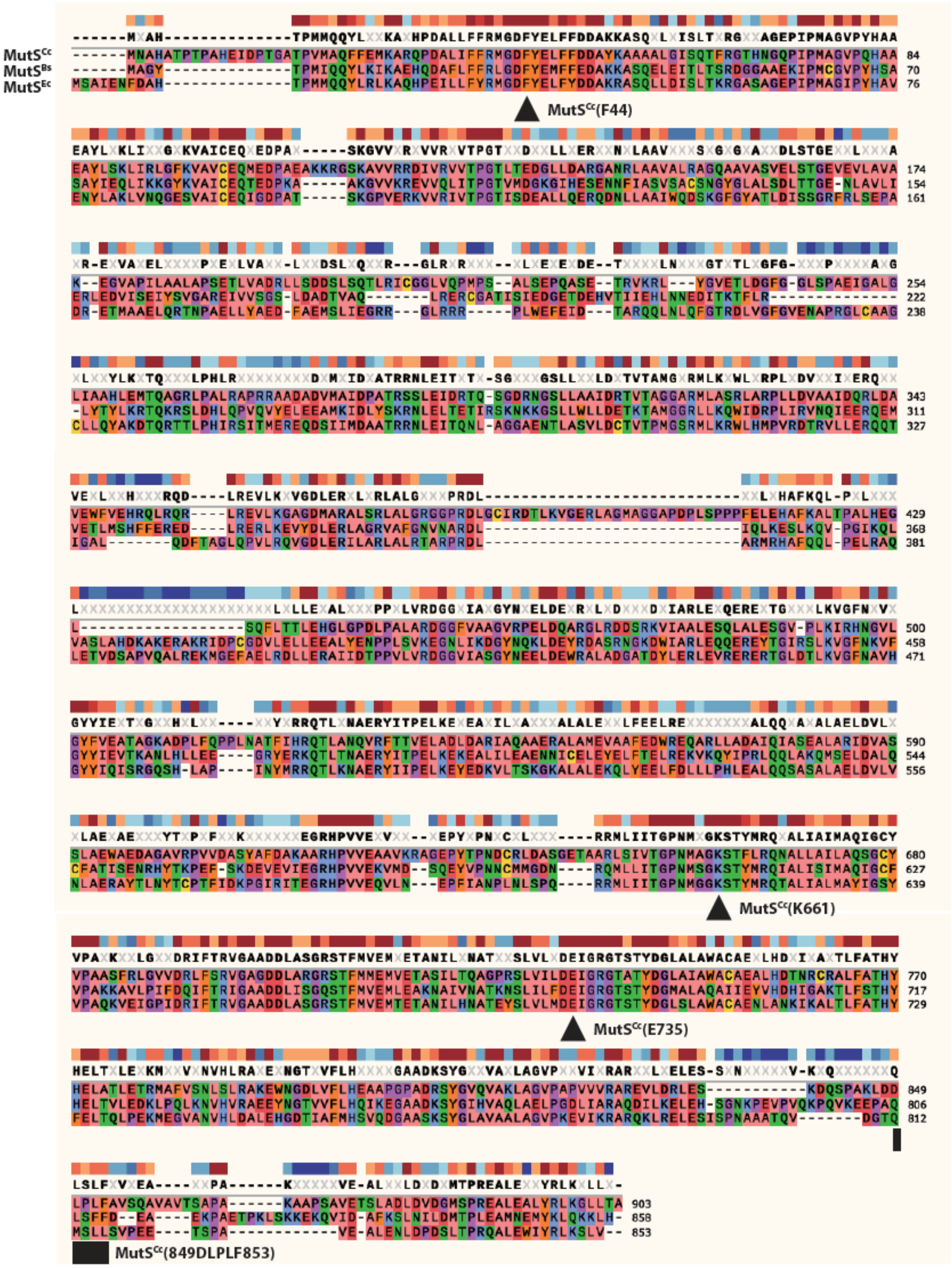

Figure S1: Similarities between selected MutS proteins. Amino-acid sequences of MutS homologs were downloaded from NCBI for *Caulobacter crescentus* NA1000 strain (MutS^{Cc}), *Bacillus subtilis* PY79 strain (MutS^{Bs}) and *Escherichia coli* MG1655 strain (MutS^{Ec}). Alignments were done using the SnapGene software (from Insightful Science; available at

snapgene.com). MutS^{Cc} residues/motifs of particular interest are indicated under the three sequences. The F44 residue belongs to a ${}_{42}$GDFYELFFDDA${}_{52}$ motif where underlined residues are part of the consensus motif for mismatch recognition by MutS proteins (4). The K661 residue belongs to a ${}_{655}$GPNMAGKS${}_{662}$ Walker A motif supposedly involved in ATP/ADP binding and whose consensus is GxxxxGK[T/S] (x can be any amino acid) (5). The E735 residue belongs to a ${}_{689}$LVLMDE${}_{694}$ Walker B motif supposedly involved in ATP hydrolysis and whose consensus is hhhhDE (h is a hydrophobic amino acid) (5). The ${}_{849}$DLPFL${}_{853}$ motif shows similarities with the β-clamp binding motifs MutS^{Bs} (${}_{806}$QLSFF${}_{810}$) (6)and MutS^{Ec} (${}_{812}$QMSLL${}_{816}$) (7).

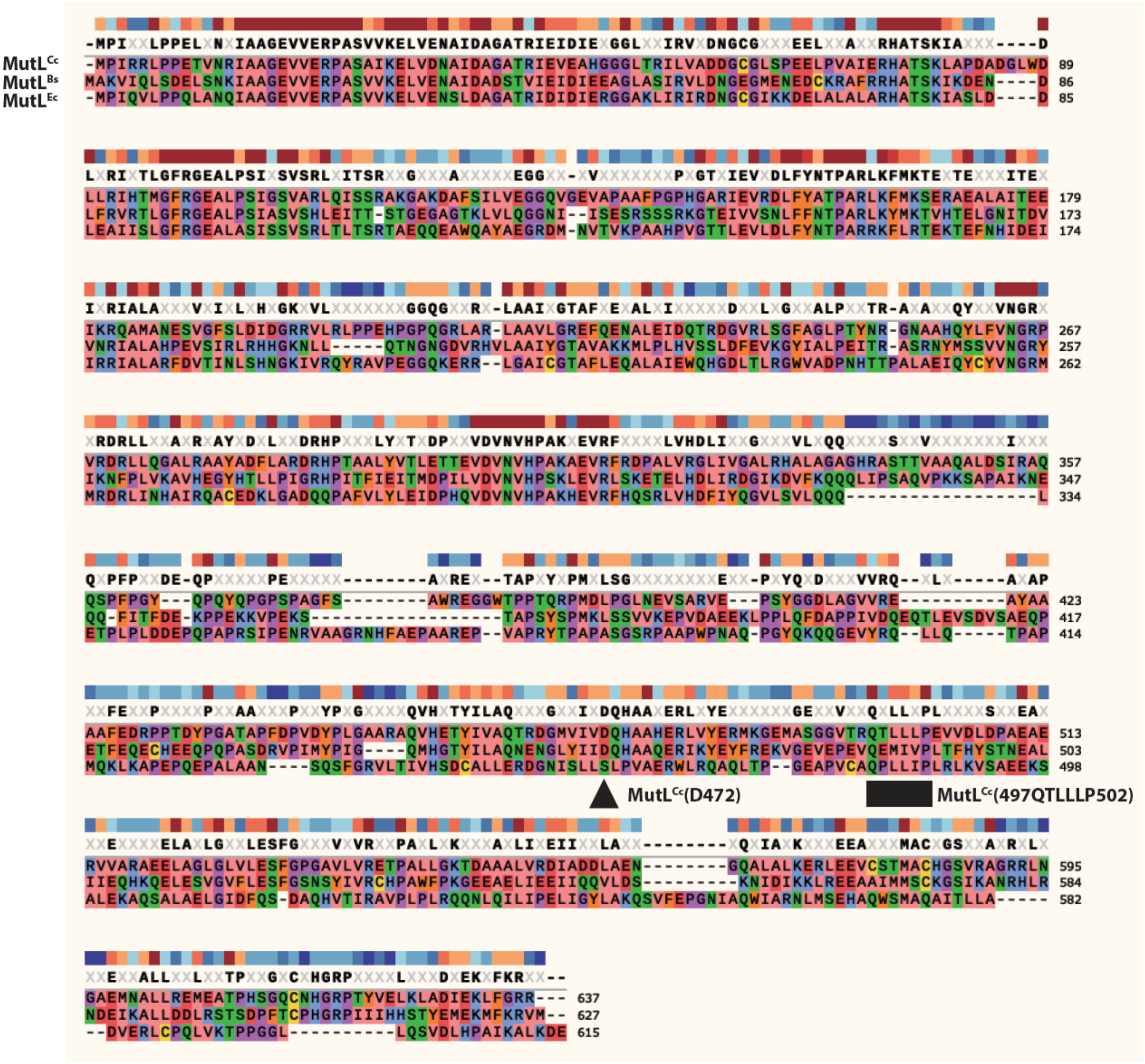

Figure S2: Similarities between selected MutL proteins. Amino-acid sequences of MutL homologs were downloaded from NCBI for *Caulobacter crescentus* NA1000 strain (MutS^{Cc}), *Bacillus subtilis* PY79 strain (MutS^{Bs}) and *Escherichia coli* MG1655 strain (MutS^{Ec}). Alignments were done using the SnapGene software. MutL^{Cc} residues/motifs of particular interest are indicated under the three sequences. The D472 residue belongs to the predicted $_{472}$DQHAAHERLVYE$_{483}$ endonuclease domain (underlined letters indicate conserved residues according to (8)). The $_{497}$QTLLLP$_{502}$ motif shows similarities with the previously proposed Qxh(L/I)xP consensus β-clamp binding motif for MutL proteins (9).

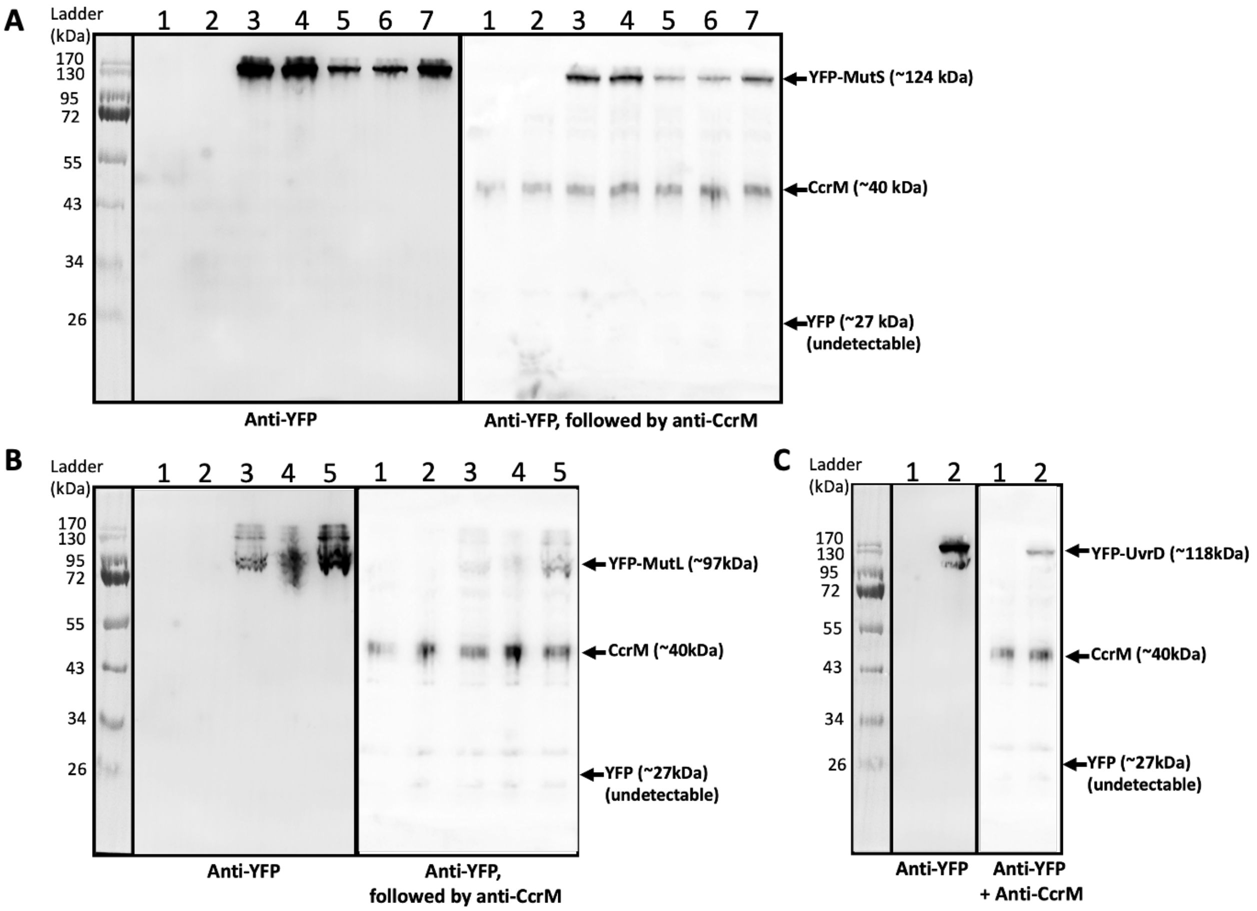

Figure S3: Immunoblot detection of YFP-tagged proteins from *C. crescentus* cell extracts. (A) Detection of YFP-tagged MutS derivatives. Cells from JC1784 (*YFP-mutS*) (lane 1) and JC1433 (Δ*mutS Pxyl::YFP-mutS*) (lane 2) cultivated into M2G medium and cells from JC1433 (lane 3), JC1666 (Δ*mutS Pxyl::YFP-mutS(F44A)*) (lane 4), JC1770 (Δ*mutS Pxyl::YFP-mutS(*$_{849}$*AAAAA*$_{853}$*)*) (lane 5), JC1665 (Δ*mutS Pxyl::YFP-mutS(K661M)*) (lane 6) and JC1739 (Δ*mutS Pxyl::YFP-mutS(E735A)*) (lane 7) were cultivated into M2G + xylose 0.3% medium. **(B) Detection of YFP-tagged MutL derivatives**. Cells from JC1769 (*YFP-mutL*) (lane 1) and JC1825 (Δ*mutL Pxyl::YFP-mutL)* (lane 2) cultivated into M2G medium and cells from JC1825 (lane 3), JC1667 (Δ*mutL Pxyl::YFP-mutL(D472N)*) (lane 4), JC1749 (Δ*mutL Pxyl::YFP-mutL(*$_{497}$*ATLAAP*$_{502}$*)*) (lane 5) were cultivated into M2G + xylose 0.3% medium. **(C) Detection of the YFP-tagged UvrD protein.** Cells from JC1870 (Δ*uvrD Pxyl::YFP-uvrD)* were cultivated into M2G (lane 1) or M2G + xylose 0.3% (lane 2) media. For all blots, proteins were separated by SDS-PAGE and YFP-tagged proteins were detected by immunoblotting using anti-GFP antibodies. The same blots were then hybridized a second time with anti-CcrM antibodies to detect CcrM (a DNA methyltransferase) as a loading control. This figure shows that the YFP moiety of each fusion protein is not cleaved off and shows the relative level of each construct.

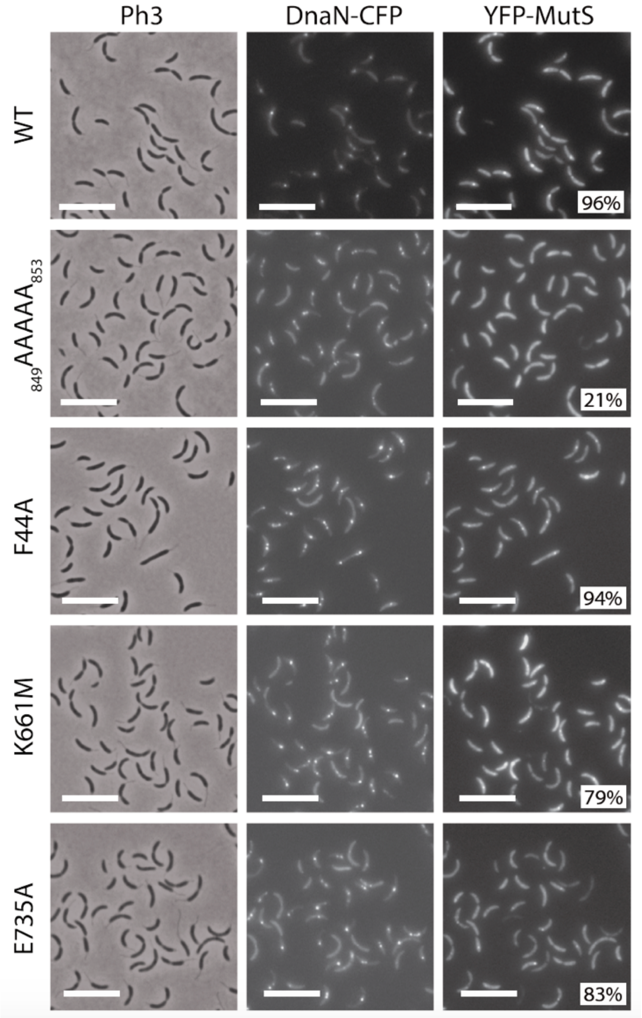

Figure S4: YFP-MutS foci co-localize with the replisome in *C. crescentus*. Subcellular localization of DnaN-CFP and derivatives of YFP-MutS in Δ*mutS* cells. Strains JC1625 (Δ*mutS dnaN-CFP Pxyl::YFP-mutS*), JC1771 (Δ*mutS dnaN-CFP Pxyl::YFP-mutS(*$_{849}$*AAAAA*$_{853}$*)*), JC1669 (Δ*mutS dnaN-CFP Pxyl::YFP-mutS(F44A)*), JC1668 (Δ*mutS dnaN-CFP Pxyl::YFP-mutS(K661M)*) and JC1740 (Δ*mutS dnaN-CFP Pxyl::YFP-mutS(E735A)*) were cultivated into PYE medium and then transferred into M2G medium. 0.3% xylose was added to cultures when they reached an OD_{660nm}~0.3. Cells were then imaged by fluorescence microscopy when the OD_{660nm} reached ~0.5. The % indicated onto images corresponds to the average proportion of distinct MutS-YFP foci (intensity >2-fold above background) that are co-localized with DnaN-CFP foci (using values obtained from three independent experiments). The white scale bar corresponds to 8μm.

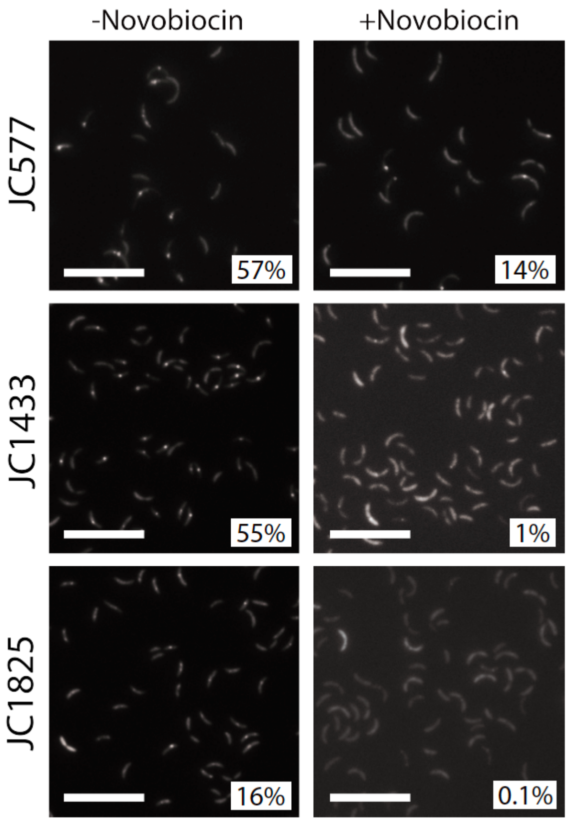

Figure S5: Replication inhibition following novobiocin treatment largely dissociates DnaN-CFP, YFP-MutS and YFP-MutL foci. Strains JC577 (*dnaN-CFP*), JC1433 (Δ*mutS Pxyl::YFP-mutS*) and JC1825 (Δ*mutL Pxyl::YFP-mutL*) were cultivated in PYE medium and then transferred into M2G medium. When the OD_{660nm} reached ~0.3, cultures were split into two and 100 μg/mL of novobiocin was added into one of the sub-cultures. 0.3% xylose was added at the same time into each sub-cultures of strains JC1433 and JC1825. Once the OD_{660nm} of the sub-culture without novobiocin reached 0.5, cells were imaged by fluorescence microscopy using CFP (for DnaN-CFP) or YFP (for YFP-MutS or YFP-MutL) illumination. Representative images are shown here. % indicated under each image correspond to the average proportion of cells displaying CFP or YFP foci (intensity >2-fold above background) as counted during three independent experiments. The white scale bar corresponds to 11μm.

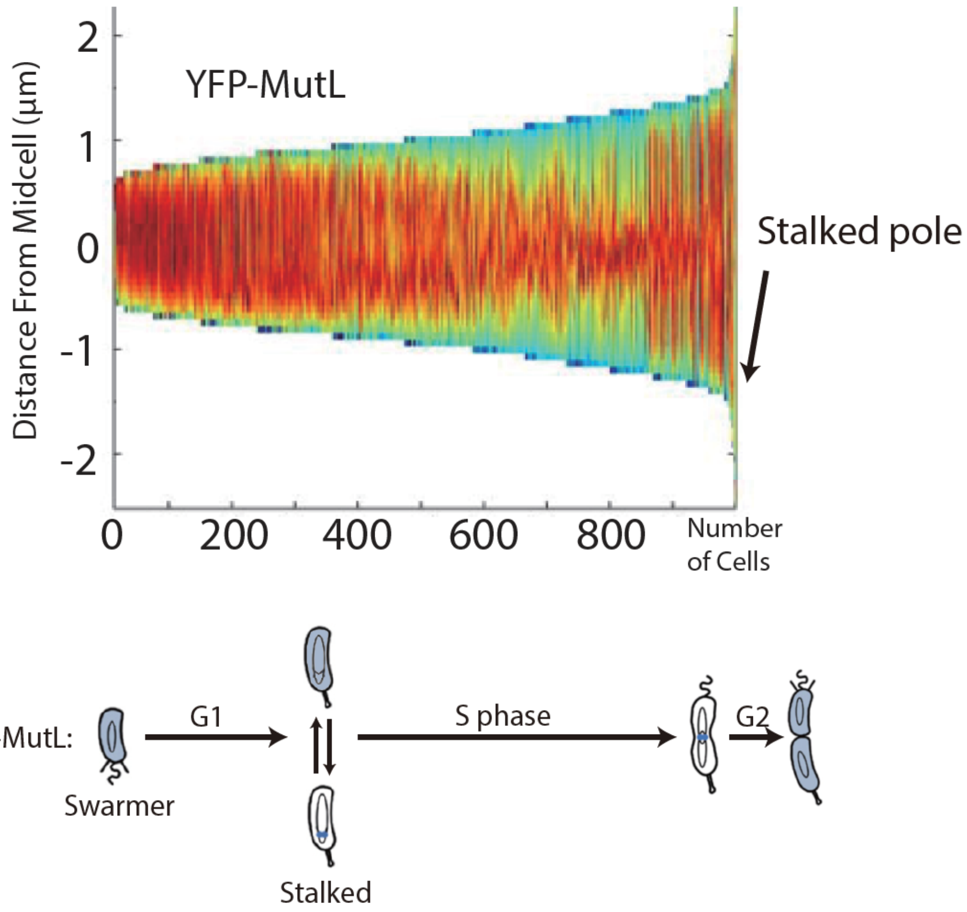

Figure S6: YFP-MutL is enriched at mid-cell towards the end of the S-phase of the *C. crescentus* cell cycle. Demograph showing the subcellular localization of YFP-MutL in Δ*mutL* cells sorted as a function of their size. JC1825 (Δ*mutL Pxyl::YFP-mutL*) cells were cultivated and imaged as described for Fig.5A. Short cells correspond to G1/swarmer cells, while intermediate and longer cells correspond to stalked and pre-divisional S-phase cells, respectively. The schematic under the demograph shows the *C. crescentus* cell cycle and the blue color highlights where YFP-MutL seems to be localized as a function of the cell cycle.

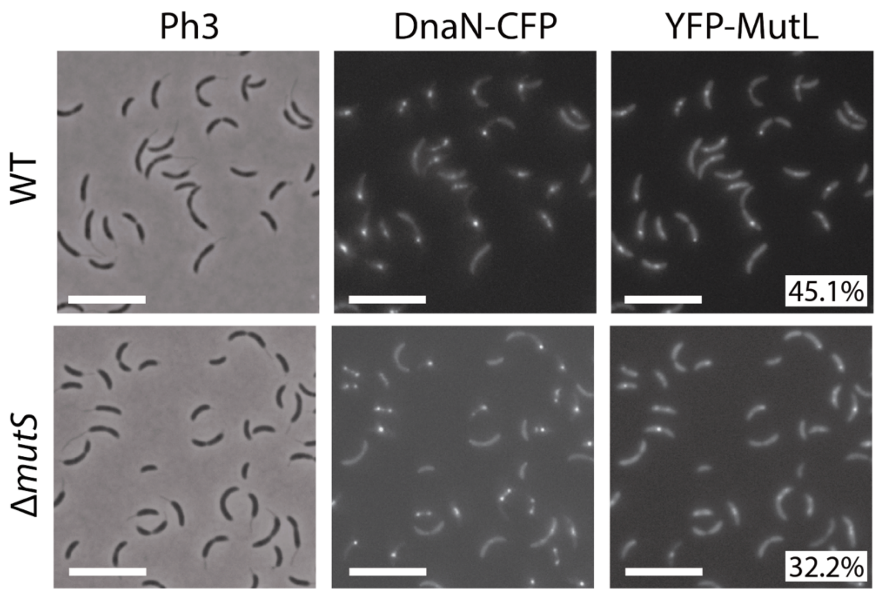

Figure S7: YFP-MutL is recruited to the replisome independently of MutS. Subcellular localization of DnaN-CFP and YFP-MutL in Δ*mutL* cells with or without *mutS*. Strains JC1812 (*dnaN-CFP* Δ*mutL Pxyl::YFP-mutL*) labelled "WT" and JC1805 (*ΔmutS* Δ*mutL dnaN-CFP Pxyl::YFP-mutL*) labelled *"ΔmutS*" were cultivated into PYE medium and then transferred into M2G medium. 0.3% xylose was added to cultures when they reached an OD_{660nm}~0.3. Cells were then imaged by fluorescence microscopy when the OD_{660nm} reached ~0.5. The % indicated onto images corresponds to the average proportion of cells with distinct YFP-MutL foci (intensity >2-fold above average background) using values obtained from three independent experiments. The white scale bar corresponds to 8μm.

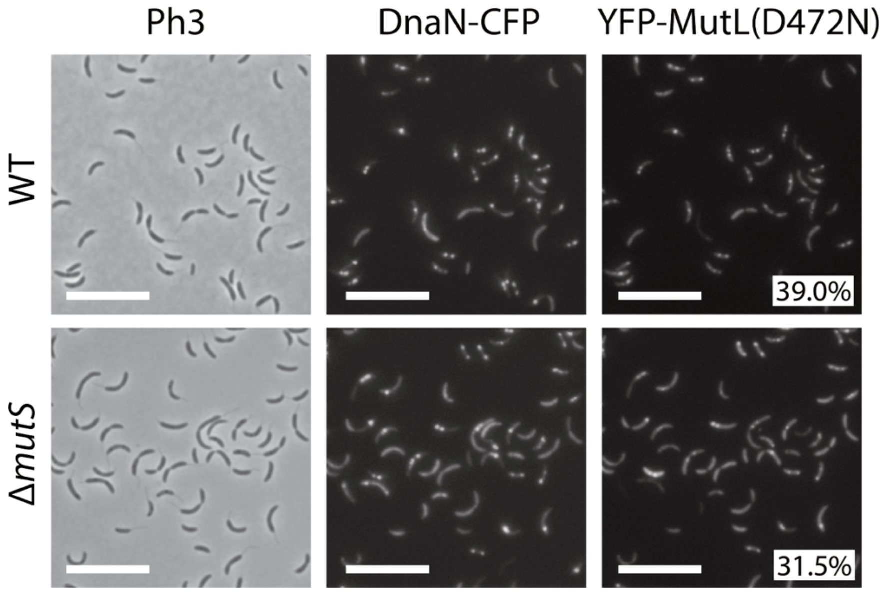

Figure S8: YFP-MutL(D472N) is stabilized at the replisome even in the absence of MutS. Subcellular localization of DnaN-CFP and YFP-MutL(D472N) in Δ*mutL* cells with or without *mutS*. Strains JC1670 (Δ*mutL dnaN-CFP Pxyl::YFP-mutL(D472N)*) and JC1894 (*ΔmutS ΔmutL dnaN-CFP Pxyl::YFP-mutL(D472N)*) were cultivated into PYE medium and then transferred into M2G medium. 0.3% xylose was added to cultures when they reached an OD_{660nm}~0.3. Cells were then imaged by fluorescence microscopy when the OD_{660nm} reached ~0.5. The % indicated onto images corresponds to the average proportion of cells with distinct YFP-MutL(D472N) foci (intensity >2-fold above average background) using values obtained from three independent experiments. The white scale bar corresponds to 11μm.

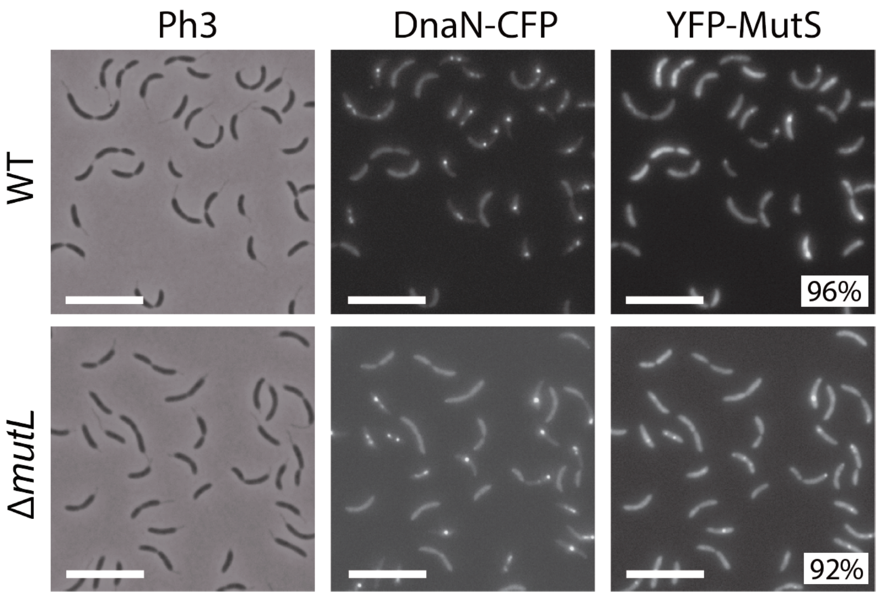

Figure S9: YFP-MutS is slightly less frequently associated with the replisome in the absence of MutL. Subcellular localization of DnaN-CFP and YFP-MutS in Δ*mutS* cells with or without *mutL*. Strains JC1625 (*dnaN-CFP* Δ*mutS Pxyl::YFP-mutS*) labelled "WT" and JC1760 (*ΔmutS dnaN-CFP Pxyl::YFP-mutS ΔmutL*) labelled *"ΔmutL*" were cultivated into PYE medium and then transferred into M2G medium. 0.3% xylose was added to cultures when they reached an OD_{660nm}~0.3. Cells were then imaged by fluorescence microscopy when the OD_{660nm} reached ~0.5. The % indicated onto images corresponds to the average proportion of distinct MutS-YFP foci (intensity >2-fold above background) that are co-localized with DnaN-CFP foci (using values obtained from three independent experiments). The white scale bar corresponds to 8μm.

Supplementary Material and Methods

Plasmids constructions

Derivatives of pNPTS138:

To construct pNPTS138-ΔmutS, pNPTS138-ΔmutL and pNPTS138-ΔuvrD, ~500 bp sequences upstream and downstream of the corresponding ORF were amplified by PCR from wild-type *C. crescentus* chromosomal DNA using the primer pairs listed in Table S1. The two fragments were subsequently digested by restriction enzymes (as indicated in Table S1) and cloned into the pNPTS138 suicide vector between corresponding restriction sites by triple ligation.

To construct pNPTS138-DnaQ(G13E), pNPTS138-MutS($_{849}$AAAAA$_{853}$) and pNPTS138-MutL($_{497}$ATLAAP$_{503}$), overlap extension PCR were used to construct ~1 kb gene fragment with the required mutations in *dnaQ*, *mutS* or *mutL*. The PCR consisted of two consecutive PCR reactions, with four primers (listed in Table S1).

To construct pNPTS138-PmutS-YFP-mutS and pNPTS138-PmutL-YFP-mutL, two primer pairs (listed in Table S1) were used to amplify the ~500 bp promoter regions (PmutS and PmutL, respectively) and the protein fusion regions (YFP-mutS and YFP-mutL, respectively). The two fragments were subsequently digested by the indicated restriction enzymes (Table S1) and cloned into the pNPTS138 suicide vector between corresponding restriction sites by triple ligation.

Derivatives of pXYFPN4:

To construct pXYFPN4-MutS, pXYFPN4-MutL and pXYFPN4-UvrD, the corresponding ORFs were amplified by PCR from wild-type *C. crescentus* chromosomal DNA using the primer pairs listed in Table S1, digested by the indicated restriction enzymes and cloned into pXYFPN4.

To construct pXYFPN4-MutS(F44A), pXYFPN4-MutS(K661M), pXYFPN4-MutS(E735A), pXYFPN4-MutS($_{849}$AAAAA$_{853}$), pXYFPN4-MutL(D472N), pXYFPN4-MutL($_{497}$ATLAAP$_{502}$) and pXYFPN4-MutL(D472N , $_{497}$ATLAAP$_{502}$) overlap PCR was used to construct a point mutation in the *mutS* or *mutL* genes. Two consecutive PCR reactions were performed using four primers (listed in Table S1). First, two DNA fragments were amplified from plasmid pXYFPN4-mutS (for *mutS* alleles) or pXYFPN4-mutL (for *mutL* alleles). Then, equimolar amounts of both products were mixed and amplified. The resulting fragments were digested by restriction enzymes (as indicated in Table S1) and cloned into the pXYFPN4 vector.

Strains constructions

Three categories of strains were constructed: (1) gene deletions or gene replacements were constructed using pNPTS138-derived plasmids (listed in Table S2) via two homologous recombination events: first, the suicide vectors were integrated at the desired location into the genome via homologous recombination and selection for kanamycin resistance. Excision of the suicide vector via a second homologous recombination event was then selected by plating on sucrose (0.3%)-containing PYEA plates and the isolated colonies were subsequently selected for kanamycin sensitivity. The mutants were identified by PCR using primer pairs used to construct pNPTS138 derivatives (Table S1) or by DNA sequencing for gene replacements; (2) pXYFPN4 derivatives were integrated at the native *xylX* locus by transformation. Positive clones were screened using RecUni-1 and RecXyl-2 primers (1). These strains were used for the inducible expression of MutS, MutL, UvrD derivatives in the presence of 0.3% xylose; (3) In JC1724, JC1845 and JC2211, the pNPTS138-DnaQ-G13E plasmid was integrated at the native *dnaQ* locus. The resulting strains express the *dnaQ(G13E)* allele together with a truncated and inactive *dnaQ*' allele. Oligos TC34 and M13-F were used to check the integration sites of the plasmid.

Immunoblot analysis

Cells were cultivated overnight in PYE medium before cultures were diluted into M2G medium. Once the OD_{660nm} reached ~0.3 (mid-exponential phase), 0.3% xylose was added when indicated. Cell extracts were then prepared when cultures reached an OD_{660nm} of ~0.5 (exponential phase) and YFP-tagged proteins were resolved on 10% SDS-PAGE gels. Proteins were then transferred to PVDF membranes (Millipore). These blots were first hybridized with anti-GFP antibodies (Roche 11814460001) diluted 1:2000 and HRP conjugated anti-mouse IgG (Sigma A9044) diluted 1:160000 and then hybridized a second time with anti-CcrM antibodies diluted 1:2000 and HRP-conjugated anti-rabbit (Sigma RABHRP1) diluted 1:30000 after a second blocking of the membrane using standard immunoblotting procedures. The signal was visualized using a chemiluminescent reagent (Amersham ECL Prime Western Blotting Detection Reagent, GE) and a FUSION FX (VILBER) scanner. Images were processed by Photoshop.

Supplementary References

1. Thanbichler, M., Iniesta, A.A. and Shapiro, L. (2007) A comprehensive set of plasmids for vanillate- and xylose-inducible gene expression in *Caulobacter crescentus*. *Nucleic Acids Res*, **35**, e137.
2. Evinger, M. and Agabian, N. (1977) Envelope-associated nucleoid from *Caulobacter crescentus* stalked and swarmer cells. *J Bacteriol*, **132**, 294-301.
3. Fernandez-Fernandez, C., Grosse, K., Sourjik, V. and Collier, J. (2013) The beta-sliding clamp directs the localization of HdaA to the replisome in *Caulobacter crescentus*. *Microbiology*, **159**, 2237-2248.
4. Lenhart, J.S., Sharma, A., Hingorani, M.M. and Simmons, L.A. (2013) DnaN clamp zones provide a platform for spatiotemporal coupling of mismatch detection to DNA replication. *Mol Microbiol*, **87**, 553-568.
5. Hanson, P.I. and Whiteheart, S.W. (2005) AAA+ proteins: have engine, will work. *Nat Rev Mol Cell Biol*, **6**, 519-529.
6. Simmons, L.A., Davies, B.W., Grossman, A.D. and Walker, G.C. (2008) Beta clamp directs localization of mismatch repair in *Bacillus subtilis*. *Mol Cell*, **29**, 291-301.
7. Lopez de Saro, F.J., Marinus, M.G., Modrich, P. and O'Donnell, M. (2006) The beta sliding clamp binds to multiple sites within MutL and MutS. *J Biol Chem*, **281**, 14340-14349.
8. Pillon, M.C., Lorenowicz, J.J., Uckelmann, M., Klocko, A.D., Mitchell, R.R., Chung, Y.S., Modrich, P., Walker, G.C., Simmons, L.A., Friedhoff, P. *et al.* (2010) Structure of the Endonuclease Domain of MutL: Unlicensed to Cut. *Mol Cell*, **39**, 145-151.
9. Pillon, M.C., Miller, J.H. and Guarne, A. (2011) The endonuclease domain of MutL interacts with the beta sliding clamp. *DNA Repair*, **10**, 87-93.
